# Modular Snake-like Robot Designed for On-Site Reconfiguration in Space Exploration

**DOI:** 10.3390/biomimetics10050293

**Published:** 2025-05-06

**Authors:** Ning Zhao, Sikai Zhao, Tianjiao Zheng, Jian Qi, Zhiyuan Yang, Xin Sui, Kai Han, Hang Luo, Nanlin Zhou, Jie Zhao, Yanhe Zhu

**Affiliations:** State Key Laboratory of Robotics and System, Harbin Institute of Technology, Harbin 150001, China; zhaoning1995@hit.edu.cn (N.Z.); zhengtj@hit.edu.cn (T.Z.); 20b908059@stu.hit.edu.cn (J.Q.); yzy19962@live.com (Z.Y.); suixinhit@163.com (X.S.); 22s108252@stu.hit.edu.cn (K.H.); 24s108234@stu.hit.edu.cn (H.L.); 23s008076@stu.hit.edu.cn (N.Z.); jzhao@hit.edu.cn (J.Z.)

**Keywords:** modular robot, snake-like motion, motion analysis, path planning, space exploration

## Abstract

Research on modular robots for space exploration has primarily focused on reconfiguration, with limited attention given to the maneuverability in space environment, which is essential for harnessing the advantages of reconfiguration. In this paper, a modular snake-like robot (MSR) is designed, which is expected to emulate a snake to navigate complex environments and employ the reconfiguration capability for on-site shape-shifting. To this end, a snake-like motion analysis and planning method is proposed for MSR. Firstly, we explore the feasibility of utilizing modules in realizing snake-like motion, including functional compatibility and structural constraints. Secondly, we analyze the kinematics of MSR and design joint coordination motion schemes to meet the requirements of snake-like motion. Finally, a path planning method based on reinforcement learning is proposed, which fully considers the motion characteristics and the structural constraints. Through motion analysis and planning, a balance between environmental adaptability and versatility can be achieved. Simulations of comparisons and potential applications further demonstrate the significant advantages of MSR in space exploration.

## 1. Introduction

With the development of aerospace technology, space exploration imposes increased requirements on robotics. Traditional robots [1,2,3,4] find it difficult to cope with the complex environments and diverse tasks in space due to their fixed structure and single function. Consequently, there is a growing interest in the field of modular robots. Composed of multiple operable and detachable modules, modular robots [5,6,7,8] can form robots with various functions and structures by redeploying the modules, holding great potential to emerge as a promising key technology in the aerospace field.

Research on modular space robots has achieved significant progress, but the related results mainly focus on module design [9,10], reconfiguration planning [11,12], and operation control [13,14], ignoring the maneuverability of modular robots in space. If the robots cannot effectively reach the task area, the advantage of reconfiguration would be greatly reduced. In weightless environments, all traditional friction-driven motions are ineffective, including rolling, crawling, and walking. At the same time, the harsh space environment also limits the possibility of the manual deployment of robots. Among the existing solutions, the method of manipulator-assisted position transfer is inefficient and difficult to navigate in complex environments. The solution of a mobile auxiliary interface lacks flexibility, cannot adapt to various tasks, and consumes resources. Flying motion [15,16] is a novel motion mode in space. It is efficient and demonstrates certain obstacle avoidance and transportation capabilities, but it needs to meet the constraints of the initial speed and can only work in a specific environment. Therefore, it is necessary to conduct in-depth research on the maneuverability of modular robots in space.

Snake-like motion is often utilized by robots to enhance their environmental adaptability due to their natural size advantages and flexibility [17,18,19]. Continued research has resulted in substantial advancements. A snake-like robot was presented in [20], capable of autonomous exploration in complex icy terrains. A robot inspired by the inchworm was proposed in [21], which exhibits superior mobility speed and load capacity. An EHD robotic finger was introduced in [22], achieving precise and flexible movement capabilities through mathematical modeling. However, the motion of these robots relies on contact forces such as friction, making them ineffective in zero-mass environments. We note that there exists a special motion mode [23,24] which uses a propulsion device to suspend the robot mid-air and generate a controllable propulsion speed. Then, the snake-like robots can use redundant degrees of freedom (DOFs) to navigate in 3D space, which can meet the requirements of maneuverability in space. Combining this motion with modular robots would enable on-site and on-demand reconfiguration, which is of significant importance for the further application of modular robots in space exploration. However, robots that realize the special snake-like motion [25,26] are designed by utilizing the function-driven approach, specifically tailored to fulfill the requirements of the special snake-like motion. Hence, the structural constraints are minimal, and the primary limitation is the joint deflection angle constraint imposed by the rigid links [27]. In contrast, modular robots are designed based on the requirements of reconfiguration, making the basic functions less compatible with snake-like motion. Additionally, modular designs may introduce additional structural constraints, further complicating motion planning and application. Therefore, it is necessary to analyze and plan according to the structure and function of the module to achieve this special snake-like motion.

**Figure 1:**
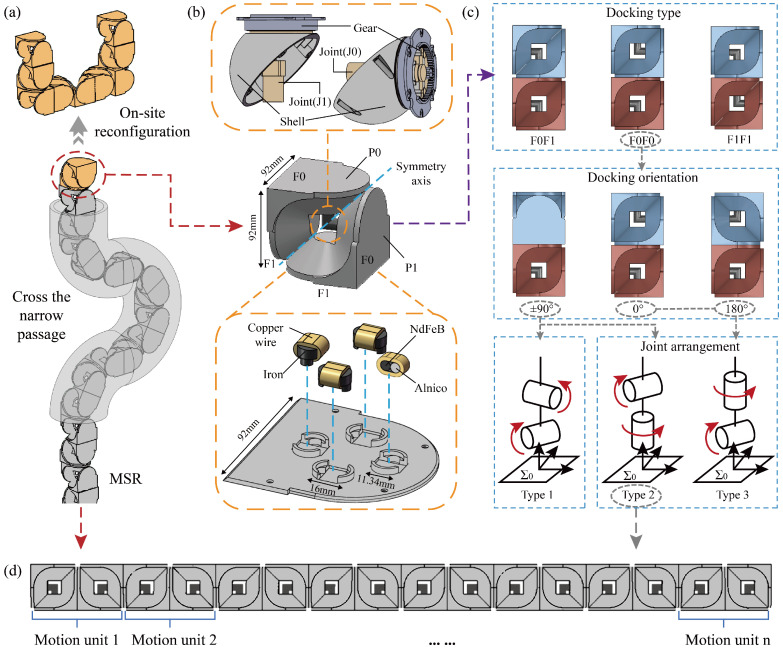
Conceptual diagram, module and structure of MSR. (**a**) Conceptual diagram. MSR travels through a narrow passage to reach the target position, and then reconfigures itself into a gripper to perform the assigned task. (**b**) Subot module. The upper figure shows the improved right-angle axis structure; the middle figure shows the basic module structure; and the lower figure illustrates the docking device structure. (**c**) The docking types, docking orientations, and joint arrangement types of the modules. (**d**) The overall structure of the MSR.

In this paper, we designed a modular snake-like robot (MSR) based on the novel space module Subot and conducted motion analysis and planning. Firstly, we analyzed the structure and function of Subot, and we systematically compared them with the principles of snake-like motion. In this way, the structure of the MSR is obtained and the structural constraints are investigated. Secondly, we explored the mechanism of propulsion speed generation and the kinematics of the MSR, and then, suitable joint coordination motion schemes were derived to ensure precise alignment with the planned path. Finally, we proposed an efficient path planning method based on reinforcement learning, which comprehensively considers task requirements, motion characteristics, and structural constraints. Through the motion analysis and planning method, MSR can utilize snake-like motion to reach the target position and complete on-site reconfiguration, achieving an organic combination of environmental adaptability and versatility. A conceptual diagram is shown in Figure 1a. Simulation comparisons demonstrated the superiority of the proposed method in terms of feasibility, efficiency, and optimization. Simulations of potential applications further showcased how MSR provide a novel working way for space exploration, paving the way for research on future robot design and planning methodologies.

## 2. Structure and Feasibility Analysis of MSR

In this section, we systematically explored the feasibility of realizing snake-like motion based on the module Subot. In the way, we obtained the structure of the MSR and the corresponding structural constraints.

### 2.1. Subot

Subot is a module for space exploration, developed based on our previously work [28,29]. It can be equivalently represented as a cubic structure composed of two L-shaped components (P0 and P1) and a right-angle axis (J0 and J1), as shown in the middle panel of Figure 1b. Among them, P0 and P1 provide four docking surfaces, which form the foundation of configurational diversity. J0 and J1 can drive P0 and P1 to rotate independently or simultaneously within a range from $-90^{\circ }$ to $+90^{\circ }$, ensuring coordinated motion and flexible operation. In addition, we conducted specific design modifications to address the requirements of the space environment. The details of Subot are shown in Table S1 of the Supplementary File, and the modifications are as follows:

The right-angle axis is replaced with two hemispherical devices connected together, as shown in the upper panel of Figure 1b. Each L-shaped component is equipped with a motor and a hemispherical shell. The motor is fixedly connected to the L-shaped component, while the hemispherical shell is linked to the motor via gears. The hemispherical shells of the two L-shaped components can be tightly connected. When the motor of one L-shaped component rotates, the gear transmission drives the hemispherical shell to rotate, thereby achieving the relative rotation of the other L-shaped component. This design aims to create a sealed structure to protect the internal transmission mechanisms and electrical systems, shielding them from dust and impacts and enhancing reliability and durability.

The docking devices adopt a novel electro-permanent magnet (EPM) technology, as shown in the lower panel of Figure 1b. The EPM consists of a neodymium-iron-boron (NdFeB) magnetic core, an alnico magnetic core, and two industrial pure iron ends. By applying an instant electric pulse, the polarity of the alnico core can be changed, thus switching the magnet between on and off states. This docking device provides significant advantages in space, as follows: the rapid response prevents the module from detachment in a microgravity environment; the low wear and energy-saving properties enhance the durability; and the attractive force between modules serves as a guiding mechanism to reduce the precision requirements for docking.

In addition, we integrated multiple functions into the EPM, such as sensing communication and energy sharing, effectively reducing the structural complexity of Subot. The sensing communication function leverages the mutual induction effect of the magnets to transmit pulse signals from the sending end to the receiving end, enabling the identification and transmission of information such as docking orientation and joint angles. By designing an H-bridge rectification circuit, energy sharing between modules can be achieved, ensuring bidirectional power transfer under arbitrary connection orientations. This prevents the failure of the entire robotic structure due to a single module running out of power. Furthermore, the module can act as a conductor to facilitate power transmission. Details of these functions can be found in our previous work [30].

### 2.2. Functional Compatibility

When snakes crawl in nature, their body follows the path traced by the head to avoid collisions. Therefore, snakes can be regarded as mechanisms consisting of multiple motion units connected in series. During movement, each motion unit sequentially repeats the actions of the previous unit. To ensure the flexible locomotion in 3D space, each motion unit must be capable of reaching any position within a spherical cap-shaped workspace, and the ideal joint is the universal joint [19]. Subot consist of two DOFs (J0 and J1) mounted perpendicularly, which can be treated as a universal joint. However, when the two motors rotate in the same direction, the connected L-shaped components follow the rotation, resulting in collisions. Hence, these DOFs are coupled and face constrains, expressed as follows:

(1) $$Angle_{J0}\times Angle_{J1}\geq 0$$

This constrain severely limits the workspace and restricts motion feasibility. Therefore, we proposed a decoupling solution where two modules each contribute one joint to form the universal joint in the motion unit. As a result, the impact analysis of different docking cases of the two modules is required.

The structure of Subot is symmetrical, as shown in the middle panel of Figure 1b, and the docking surfaces can be categorized into two types, F0 and F1. As a result, there are three docking types between two modules, as shown in the upper panel of Figure 1c. For each docking type, there are four docking orientations. We take F0F0 as an example, as shown in the middle panel of Figure 1c. There are a total of 12 docking cases. Since each module only provides one rotational axis and the two axes have different directions, the joint arrangement of driving and driven forks result in three types, as illustrated in the lower panel of Figure 1c. Among these, the docking orientations of $0^{\circ }$ and $180^{\circ }$ can satisfy the latter two types, while $\pm 90^{\circ }$ orientations can satisfy all three types. The joint arrangement of Type 1 is similar to a traditional snake-like robot, and the universal joint consists of a pitch joint and a yaw joint. By combining the two rotational angles, the target angle can be synthesized to fit the desired path points. Type 2 is a novel arrangement type, and the universal joint consists of a roll joint and a pitch/yaw joint. Although the two axes can generate arbitrary angles within a spherical workspace, sudden changes in joint angles may occur when the driving fork faces rotational constraints. However, when the docking orientation of Type 2 is $0^{\circ }$ and $180^{\circ }$, all modules have an axis perpendicular to the link. In 2D or special environments, each module can act as an independent motion unit to improve motion smoothness. Type 3, due to its limited workspace, is excluded from consideration.

In conclusion, different docking cases can form either joint arrangement Type 1 or Type 2. Therefore, the special snake-like motion can be achieved by the MSR. In practical applications, the docking case would depend on the task requirements. In this paper, we assume that the task requirement is using the meta-module method described in [31] to reconfigure after reaching the task position. Hence, we adopt the second docking type, the third docking orientation, and Type 2, as shown in Figure 1c. The overall structure of the MSR is illustrated in Figure 1d.

### 2.3. Structural Constrains

**Figure 2:**
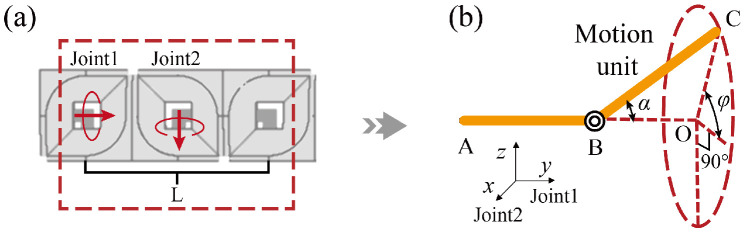
Motion unit and theoretical model. (**a**) Motion unit composed of modules. (**b**) Theoretical model of the motion unit.

As previously analyzed, the motion unit is composed of two modules. As shown in Figure 2a, the two modules provide rotational axes Joint1 and Joint2 to form a universal joint. Joint1 acts as the driving fork, while Joint2 acts as the driven fork. The link is the length between one Joint1 and the next Joint1 along the module body.

Although the motion unit can be abstracted into the theoretical model shown in Figure 2b, certain structural constraints exist in practice. The first is the joint deflection angle constraint. To ensure all joints align with the planned path, the rigid links will inevitably deviate from the path line. This deviation may result in the actual joint deflection angles departing from the smooth transition between path points, potentially exceeding the angles of the path points.

**Figure 3:**
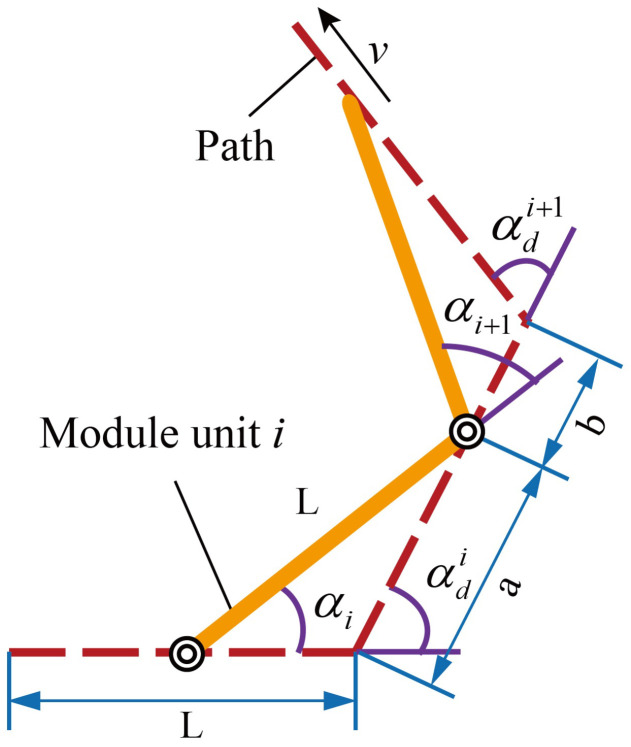
Trigonometric equivalent graph.

As shown in Figure 3, when the motion unit i+1 moves along a path line, its joint deflection angle is given by the following:

(2) $$\alpha_{i+1}=\alpha_{d}^{i}+\alpha_{d}^{i+1}-\arcsin\left(\frac{a}{L}\sin\left(\alpha_{d}^{i}\right)\right)-\arcsin\left(\frac{b}{L}\sin\left(\alpha_{d}^{i+1}\right)\right)$$

where $\alpha_{i}$ and $\alpha_{i+1}$ denote the rotation angles of the driven forks in motion units *i* and i+1, *L* represents the link length and the path line length, $\alpha_{d}^{i}$ and $\alpha_{d}^{i+1}$ are the rotation angles of the driven forks at path points *i* and i+1, and the sum of *a* and *b* equals *L*, which are utilized to represent the current position of motion unit i+1.

It can be seen that the joint deflection angle is related to the deflection angles of the path points and the position along the path line. When the directions of the path point deflection angles are consistent, the joint deflection angle would exceed the path point deflection angles. Moreover, the position along the path line where the maximum joint deflection occurs depends on the path point deflection angles, which cannot be determined precisely or calculated directly. Therefore, equidistant numerical search is required to obtain the maximum joint deflection angle between any two path points, and this angle must be compared with the joint rotation limit to avoid path deviations and hardware damage. Hence, we incorporate the calculation and comparison into the path planning by validating the feasibility of the next path point in real time.

**Figure 4:**
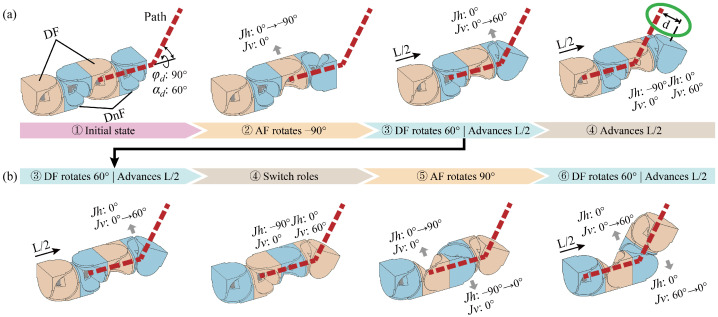
Deviation constrain and the solution. The red dashed line represents the planned path, the orange module is the driving fork module (DF), and the blue module is the driven fork module (DnF). $\varphi_{d}$ and $\alpha_{d}$ denote the path point deflection angles, Jh represents the driving fork, Jv represents the driven fork, and *L* is the distance between the path points, the green circle marks the deviation problems, with *d* representing the deviation. (**a**) The scenario where the joints fail to align with the path. (**b**) The result of employing the dynamic motion unit method.

Second, we address the deviation constrain due to the decoupling of the driving and the driven fork, which prevents the joints from aligning with the path, as shown in Figure 4a. To address this issue, we leverage the modular interchangeability to propose a dynamic motion unit method. Specifically, for every half-segment along the path line, the two modules in each motion unit are redefined as follows: the module containing the driving fork combines with the subsequent module to form a new motion unit, taking on the role of providing the driven fork, whereas the module containing the driven fork combines with the preceding module to form a new motion unit, taking on the role of providing the driving fork, as shown in Figure 4b. In this way, the MSR can ensure precise alignment with the planned path.

**Figure 5:**
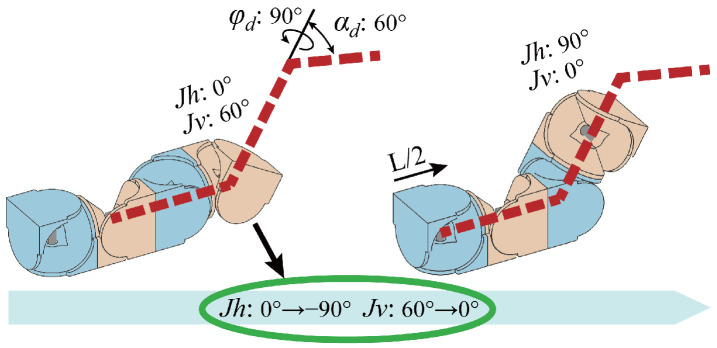
Joint coupling constrain. The first module needs to simultaneously rotate its two joints to ensure alignment with the path. However, during the rotation process, the module fails to satisfy the joint coupling constraint, resulting in motion failure. The green circle marks the coupling of module joints.

The third problem relates to the joints’ coupling constraint in Equation (1). The solution involves utilizing two modules, each contributing a rotational axis, to form the universal joint of the motion unit. Therefore, only one rotational axis within a module is rotating at each time point. However, in the dynamic motion unit method, the role transition of the module requires both joints to rotate simultaneously. If the joint coupling constraint cannot be satisfied, the motion will fail, as shown in Figure 5. Additionally, due to the rotational limitations of the driving fork, sudden changes in angles may occur when solving the target angles.

**Figure 6:**
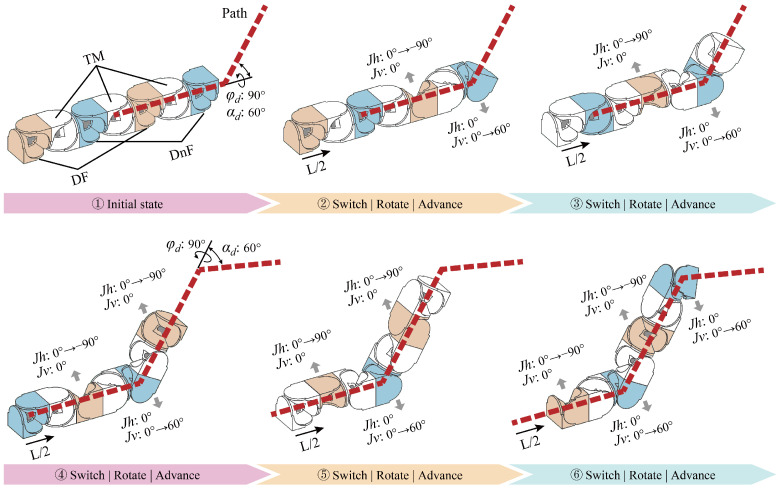
Results of adding transitional modules (TMs).

To address this issue, we introduced two transitional modules into the motion unit, placing a module in front of the driving fork module and the driven fork module, respectively. Since the dynamic motion unit method is employed, these modules do not affect the alignment between the joints and the path. During the process of role redefinition, the modules can first transition into transitional modules with zero angles before assuming the new roles, which effectively prevents joint coupling constraint, as shown in Figure 6.

Through the above solution, we resolved the structural constraints, thereby verifying the feasibility of MSR in realizing the special snake-like motion.

## 3. Joints Coordination Motion Schemes

Through the feasibility analysis, the MSR possesses the potential of achieving snake-like motion. Then it is necessary to design joint coordination motion schemes for the MSR based on the characteristics of the special snake-like motion, including propulsion speed generation and the kinematic analysis.

### 3.1. Generation of Propulsion Speed

Unlike the motion of traditional manipulators, the special snake-like motion possesses an overall propulsion to achieve continuous advancement along a planned path. Traditional approaches employ a linear sliding rail to achieve propulsion [19,32]. However, the installation of a sliding rail in a space environment is challenging, and the size of the rail would limit the scalability of the MSR. To address this issue, we proposed a fold–unfold method by leveraging the redundant DOFs in the MSR. By gradually unfolding the folding structure, an initial propulsion speed is generated, which not only achieves efficient overall propulsion but also reduces the spatial utilization efficiency.

**Figure 7:**
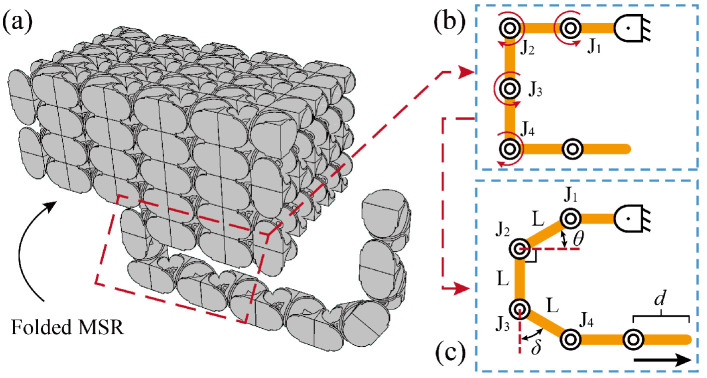
Generation of propulsion speed. (**a**) Folded MSR. (**b**) The initial state of the four joints which are utilized to generate propulsion speed. (**c**) The mechanism of generating propulsion speed through joint rotation.

As shown in Figure 7a, MSR is initially folded into a compact cubic structure. Upon receiving task instructions, the MSR utilizes the rotation of four joints to generate a speed in a specific direction, as shown in Figure 7b,c. Among these, joints J1 and J2 rotate in opposite directions with identical angles θ, joints J3 and J4 rotate in opposite directions with identical angles δ. Furthermore, J1 and J3 rotate in the same direction, and the link length is *L*. The relationship between the forward displacement *d* and the rotation angles is given as follows:

(3) $$\begin{array}{c} \delta =\arccos\left(1-\sin\left(\theta \right)\right)\\ d=L-L\cos\left(\theta \right)+L\sin\left(\delta \right) \end{array}$$

By sequentially replacing the four joints, the MSR achieves a controllable forward propulsion speed, enabling efficient motion along the planned path.

### 3.2. Kinematic Analysis

Another notable characteristic of the special snake-like motion is that all joints can be consistently aligned with the planned path during its movement [19,32], which endows it with remarkable adaptability in complex environments. In this section, we conducted a kinematic analysis of the MSR. The objective is to establish the relationship between the head movement speed, the joint angles, and the overall propulsion speed, which can ensure the precise alignment with the planned path and enhance the controllability.

Based on the theoretical model of the motion unit shown in Figure 2b, the forward and inverse kinematics are expressed as follows:

(4) $$p\left(\alpha ,\varphi ,L\right)=\left[\begin{array}{c} p_{x}\\ p_{y}\\ p_{z} \end{array}\right]=\left[\begin{array}{c} L\sin\left(\alpha \right)\sin\left(\varphi \right)\\ L\cos\left(\alpha \right)\\ L\sin\left(\alpha \right)\cos\left(\varphi \right) \end{array}\right]$$

(5) $$r\left(p_{x},p_{y},p_{z}\right)=\left[\begin{array}{c} \varphi \\ \alpha \end{array}\right]=\left[\begin{array}{c} \arcsin\left(\frac{p_{x}}{L\sin\left(\alpha \right)}\right)\\ \arccos\left(\frac{p_{y}}{L}\right) \end{array}\right]$$

where α and φ represent the rotation angles of the driven fork and the driving fork, *L* represents the link length, and $p\left(\alpha ,\varphi ,L\right)={\left[\begin{array}{ccc} p_{x} & p_{y} & p_{z} \end{array}\right]}^{T}$ denotes the coordinate position of the motion unit.

**Figure 8:**
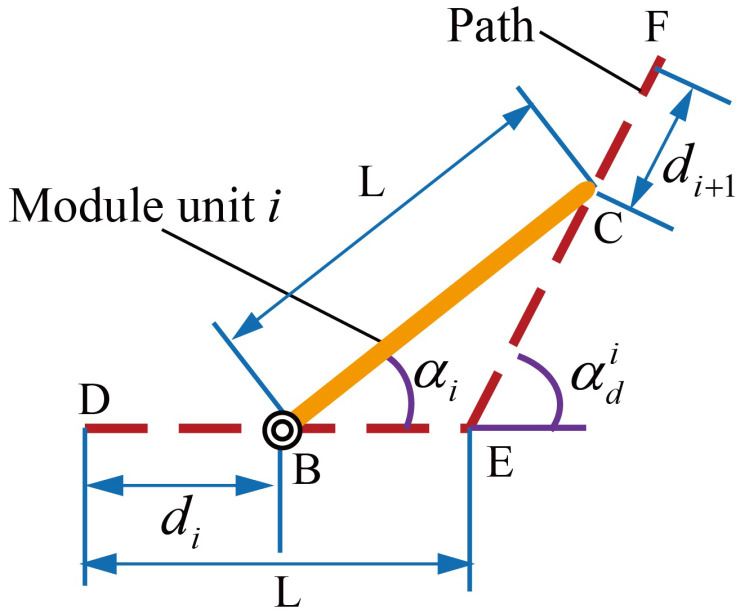
Trigonometric equivalent graph.

Due to the design of the transitional modules and the dynamic motion unit method, the driving fork and the driven fork within the motion unit are fully decoupled, and they are not influenced by neighboring motion units. Furthermore, the rotation of the driving fork is completed before the movement along the path, hence only the rotation of the driven fork needs to be considered. Taking the geometric relationship of motion unit *i* at a specific moment as an example, its trigonometric equivalent graph is shown in Figure 8, and the following relationship can be derived:

(6) dAB+dBC=LdCD+dDE=LdBCdBCsinαdi−αisinαdi−αi=dCDdCDsinαisinαi=LLsinαdisinαdi

where $d_{xy}$ represents the distance between point *x* and point *y* in Figure 8, $x,y\in \left(B,C,D,E,F\right)$. Assuming the propulsion speed is *v*, the above relationship can be extended to the entire structure as follows:

(7) $$\begin{array}{c} d_{n}=vt\\ \varphi_{i}=\varphi_{d}^{i}\\ \alpha_{i}=\arcsin\left(\frac{vt\sin\left(\alpha_{d}^{i}\right)}{L}\right)\\ d_{i-1}=L-\frac{L}{\sin\left(\alpha_{d}^{i}\right)}\cdot \sin\left(\alpha_{d}^{i}-\alpha_{i}\right) \end{array}$$

where $\alpha_{i}$ denotes the rotation angles of the driven forks in motion units *i*, *L* represents the link length and the distance between path points, and $\alpha_{d}^{i}$ represents the rotation angles of the driven forks at path points *i*.

Through Equation (7), each joint in MSR can rotate sequentially without conflicts or collisions, and all motion units remain aligned with the planned path to achieve snake-like motion. Compared to the kinematic analysis of traditional snake-like robots, the proposed method achieves complete decoupling. On the one hand, only one joint of the module is rotating at each time point, while the other joint maintains an angle of zero, which successfully avoids the joint coupling constraint of the module. On the other hand, the utilization of transitional modules solves the mutual influence of rotation between modules. Each motion unit only needs to rotate independently and follow the rotation trajectory of the previous joint at the previous time step. Thus, there is no need for path retracing or recalculation, simplifying the computation of joint angles during motion.

When the MSR moves in 2D or special environments, the transitional modules can be omitted. Because the driving forks will not rotate, the joint coupling constraint is avoided. Moreover, omitting the transitional modules can reduce the length of the links, improving the precision of motion. However, the mutual influence between motion units must be considered in this case. When calculating the joint rotation angles, it is necessary to retrace all traversed paths and use the equidistant numerical search to obtain the actual joint rotation angles. Assuming motion unit *i* is currently moving along the path toward the *m* path point, and based on previous derivations, the joint rotation angle of the motion unit along the path is already known as $\alpha_{i}$. The compensated joint rotation angle is ${\alpha_{i}}^{\prime }$, which can be calculated using the following formula:

(8) $$\begin{array}{c} p\left({\alpha_{i}}^{\prime },L\right)=\underset{2}{\underset{︸}{R_{x}\left(\alpha_{m}\right)\cdots R_{x}\left(\alpha_{1}\right)}}\\ \;\;\;\;\;\;\underset{1}{\underset{︸}{R_{x}\left(-\alpha_{d}^{0}\right)\cdots R_{x}\left(-\alpha_{d}^{m-1}\right)}}p\left(\alpha_{i},L\right) \end{array}$$

where 1 refers to the retracing of path points back to the initial coordinate frame, while 2 involves the iteration to account for the mutual influence between links, $\alpha_{d}^{0}$ is set to 0, and $R_{x}\left(\theta \right)$ represents the rotation transformation matrix for the coordinate system. Then the compensated angles can be obtained by substituting $p\left({\alpha_{i}}^{\prime },L\right)$ into Equation (5). In addition, omitting the transitional modules requires adding joint deflection angle constraints to the path planning process, which further increases the computational load. When working in 2D environments, it is necessary to comprehensively consider the trade-off between path precision and computational complexity to determine the most suitable method.

## 4. Path Planning

A suitable path is crucial for the special snake-like motion. When planning the path, it is essential not only to meet task requirements, such as obstacle avoidance, efficiency, and smoothness, but also to fully consider the motion characteristics and structural constraints to ensure the feasibility of the path and motion. Traditional path planning methods, such as geometric [32] and optimization-based planning [33], face challenges with the high-dimensional configuration space of MSR. Random sampling methods [34,35], which do not require objective function computation, demonstrate strong adaptability but are influenced by sampling distributions, potentially leading to unsmooth or infeasible paths. In contrast, learning-based methods [36,37] explore the environment through trial-and-error processes, circumventing explicit planning and showing significant advantages in multi-task operations. Therefore, this study adopts reinforcement learning and combines it with the motion characteristics, structural constraints, and task requirements to enhance applicability.

### 4.1. State-Action Design

When the MSR moves along the planned path utilizing the snake-like motion, the rotation of each motion unit in the MSR is a delayed reproduction of the head motion unit. As a result, path planning finds it sufficient to focus solely on the rotation trajectory of the head motion unit. To simplify the problem, the head motion unit is modeled as an agent that can move in any direction with a fixed step length. Therefore, the planning process is defined as follows: the agent starts from an initial position and moves straight in the current direction by a fixed length *L*, then a new direction is selected, and the agent continues to move by the same length *L* in the new direction. This process is repeated until the agent reaches the specified target position.

Thus, the motion of the agent from its current position to the next position is determined by the following three elements: the deflection angle φ of the driving fork, the deflection angle α of the driven fork, and the path line length. The direction is synthesized by the two joint angles, and the range of the joint deflection angles is −π−π22ππ22. Based on this, the action is designed as follows:

(9) $$a={\left[\begin{array}{cc} \alpha & \varphi \end{array}\right]}^{T}$$

The state is to describe the relationship between the motion of the agent and the target position. Therefore, the state consists the following aspects:

(10) $$s={\left[\begin{array}{ccc} \begin{array}{ccc} dx & dy & dz \end{array} & d\alpha & \begin{array}{cc} d\varphi & n \end{array} \end{array}\right]}^{T}$$

where dx, dy, and dz represent the coordinate differences between the current position and the target position; dα and dφ represent the deflection angles between the current position and the target position in the current coordinate frame; and *n* represents the number of steps taken by the agent.

### 4.2. Reward Design

The reward is the scalar feedback provided by the environment to the agent after it performs an action. In reinforcement learning, designing a well-crafted reward function is crucial, as it guides the agent toward an optimal policy and accelerates the convergence. To meet the requirements of snake-like motion and tasks, a reward function consisting of four components is proposed as follows:

(1) Distance is the intuitive metric for judging success. Using it as part of the reward system encourages the agent to move to the target. Euclidean distance is utilized as the reward value as follows:
  (11) $$r_{1}=\left\{\begin{array}{c} +100\;\;\;\;\;\;\;\;\;\;\;\mathrm{if}\;\mathrm{reach}\;\mathrm{the}\;\mathrm{target}\\ -\beta \sqrt{{\left(dx\right)}^{2}+{\left(dy\right)}^{2}+{\left(dz\right)}^{2}}\;\;\;\mathrm{else} \end{array}\right.$$
  where β>0 is a scaling factor to control the magnitude of the reward.
(2) Collision is strictly prohibited in path planning. To ensure the agent avoids obstacles, collision is incorporated as part of the reward function, as follows:
  (12) $$r_{2}=\left\{\begin{array}{c} -c\;\;\mathrm{if}\;\mathrm{robot}\;\mathrm{collided}\\ 0\;\;\;\;\mathrm{else} \end{array}\right.$$
  where c>0 is a large penalty value designed to emphasize the importance of avoiding collisions.
(3) In path planning, it is necessary to take joint deflection angle constraints into account to ensure the feasibility of movement. Under normal situations, the motion units are fully decoupled due to the utilization of the transitional modules and the dynamic motion unit method. Hence, the maximum joint deflection angle corresponds to the path point deflection angle, which can be constrained during action generation. However, for the 2D or special environments, it is required to utilize Equation (2) to calculate the maximum joint deflection angle $\alpha_{i}^{\max}\left(i=1,2,\cdots ,n\right)$ and use it as a reward to train the agent. Moreover, in all situations, the path point deflection angle $\alpha_{d}^{i}\left(i=1,2,\cdots ,n\right)$ is incorporated into the reward function to improve the smoothness of the path.
  (13) r3=−cif2−dandππ22<αimaxorαimax<−π−π22−λαdielse
  where λ>0 is a scaling factor used to control the magnitude of the reward.
(4) Adding the step number into the reward function can reduce the length of the planned path. On the one hand, it can reduce energy consumption and improve the efficiency, on the other hand, it helps prevent potential detours.
  (14) $$r_{4}=-kn$$
  where k>0 is a scaling factor used to control the magnitude of the reward.

In summary, the reward function can be expressed as follows:

(15) $$r=r_{1}+r_{2}+r_{3}+r_{4}$$

### 4.3. PPO Algorithm

The action space mentioned above is continuous, making it necessary to select a suitable algorithm. Proximal Policy Optimization (PPO) is a reinforcement learning algorithm specifically designed for continuous action spaces. Compared to other algorithms, PPO offers superior stability and convergence efficiency, while demonstrating excellent performance in handling high-dimensional continuous action space. As a result, it has been widely applied in the research and practice of fields such as robotic control and game agents.

The PPO algorithm consists of two networks, an Actor network and a Critic network. The Actor network is responsible for generating the policy, while the Critic network evaluates the current policy by calculating the advantage function. The objective function is expressed as follows.

(16) $$\begin{array}{c} L_{t}^{CLIP}\left(\theta \right)=\sum_{\left(st,at\right)}min\left(r_{t}\left(\theta \right)A\left(s_{t},a_{t}\right),\right.\\ \;\;\;\;\;\;\left.clip\left(r_{t}\left(\theta \right),1-\varepsilon ,1+\varepsilon \right)A\left(s_{t},a_{t}\right)\right) \end{array}$$

where ε is the clipping coefficient, θ represents the parameters of the Actor network, and $r_{t}\left(\theta \right)$ denotes the ratio between the new policy and the old policy, as follows:

(17) $$r_{t}\left(\theta \right)=\frac{\pi_{\theta }\left(a_{t}|s_{t}\right)}{\pi_{\theta old}\left(a_{t}|s_{t}\right)}$$

Variable $A\left(s_{t},a_{t}\right)$ represents the advantage function, which can reduce variance as much as possible while remaining unbiased. The expression is as follows:

(18) $$A\left(s_{t},a_{t}\right)=Q\left(s_{t},a_{t}\right)-V\left(s_{t}\right)$$

During the update process, the PPO algorithm utilizes Equation (16) to limit the update magnitude of the policy. When the offset between the new and old policies becomes large, the clipping term is used as a replacement. This ensures that the deviation between the new and old policies remains within a reasonable range, enabling the Actor network to update in a relatively stable manner and accelerating the convergence speed. Specifically, the Actor network generates the current action for the robot based on the current state. The robot then executes this action, resulting in a new state and receiving a reward, completing one full interaction process. Interaction data from multiple such episodes are stored in batches and used to update the Actor and Critic networks, ultimately obtaining relatively optimal network parameters.

## 5. Simulation Experiments

### 5.1. Simulation Comparisons

**Figure 9:**
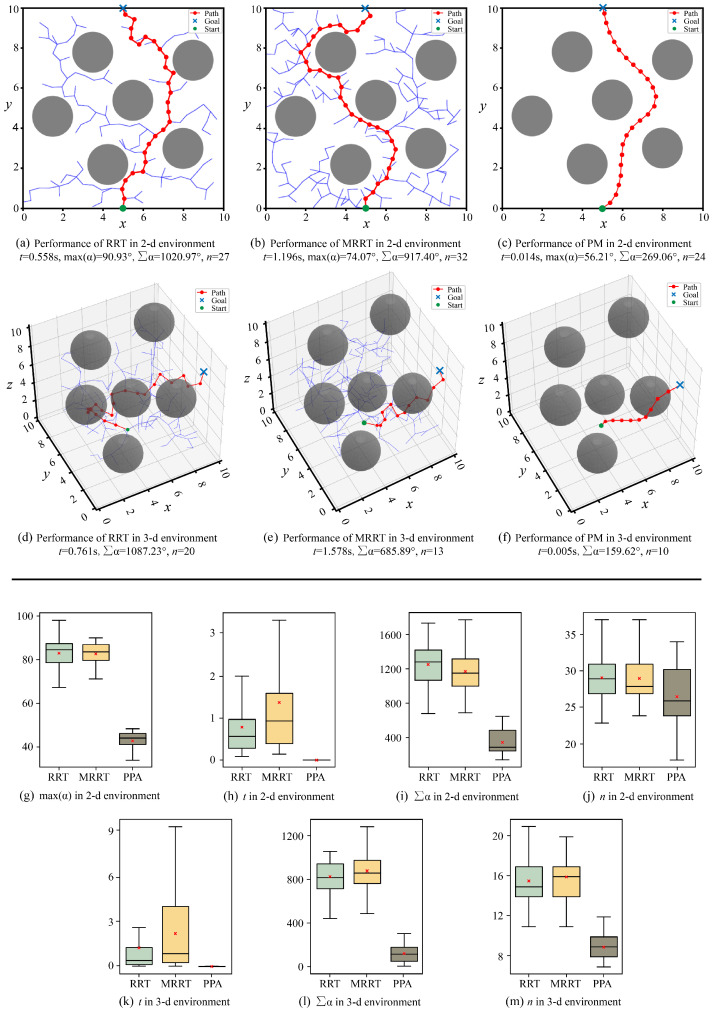
Results of the three methods. *t* is the time consumption. $\max\left(\alpha \right)$ is the maximum joint deflection angle. ∑α the total of the path deflection angles. *n* is the step number.

In the simulation experiments, the proposed method (PM) is compared with traditional methods to validate its advantages. The first method is the classical Rapidly-exploring Random Tree (RRT) method, which utilizes random sampling to rapidly expand the path tree. This method is known for its strong global search capability and high computational efficiency, making it widely adopted in various applications. The second method is the MRRT [27], which incorporates joint deflection angle constraints into the RRT to improving the feasibility of the planned paths. Notably, the universal joint utilized by the MRRT differs from the one in this study. Moreover, the MRRT simplifies the calculation of the maximum joint deflection angles by assuming that the maximum value appears in the middle position of a path line, which neglects the actual distribution of joint deflection angles. For fairness, we modified the MRRT by utilizing the universal joint type mentioned in our work and calculating the maximum joint deflection angles by equidistant numerical search. Comparative evaluations of the three methods are conducted in terms of feasibility, maximum joint deflection angles, time efficiency, and path smoothness. The simulation experiment environments include two types, 2D and 3D environments, and the results are presented in Figure 9. The simulation settings are shown in Supplementary S1.

Figure 9a–c illustrate the results of the three path planning methods in a 2D environment. All three methods successfully avoid obstacles and reach the target position. To further verify the feasibility of the methods, we conducted 100 tests for each method, and all tests are successful. Figure 9d–f presents the results of the three path planning algorithms in a 3D environment. Similar to the 2D environment, all three methods successfully reach the target position. Repeated experiments were also conducted for the 3D environment. The results reveal that the success rate of traditional algorithms decreases compared to the 2D environment. This is attributed to the increased complexity of the environment, which caused the exploration steps to exceed the predefined limit. After increasing the predefined limit, the success rate significantly improved. In contrast, the PM maintained a high success rate, as the success depends on the quality of the pre-trained model. Therefore, the proposed method demonstrates superior feasibility and reliability when dealing with complex environments. Finally, by integrating the proposed joint coordination motion schemes, we implemented the snake-like motion in both environments, further validating the feasibility of the proposed method. The results are shown in Supplementary Figures S1 and S2.

In the 2D environment shown in Figure 9a–c, the maximum joint deflection angles are calculated. In the path generated by the RRT, the joint of motion unit needs to rotate to move from two path points, which exceeds the joint deflection angle constraints. In contrast, both the MRRT and PM imposed constraints during the planning process, thus avoiding the joint deflection angle constraints. During 100 tests, we conducted a statistical analysis of the maximum joint deflection angle, as shown in Figure 9g. It is evident that the RRT performs the worst, with a certain probability of exceeding the constraint. Although the MRRT successfully avoids the constraints, its maximum joint deflection angle is larger than that of the PM. Comparatively, the PM not only avoids the constraint but also achieves smaller angle values.

As shown in Figure 9a–f, PM outperforms traditional methods in terms of time consumption. For fairness, we collected time consumption data over 100 tests, as shown in Figure 9h,k. In all environments, the PM demonstrates the best performance in terms of time efficiency. This is because the PM relies on the pre-trained model for path planning, eliminating the need for extensive calculations, with its time cost arising from the calculation of the maximum joint deflection angle. In contrast, the RRT and MRRT require significant exploration and computation, leading to higher time consumption.

As shown in Figure 9a–f, the paths planned by the PM are significantly smoother. We used two metrics to evaluate the quality of the paths, the total of the path deflection angles, and the step number. Statistical results based on 100 tests, as shown in Figure 9i,j,l,m, indicate that the PM outperforms traditional methods in both metrics. This improvement is attributed to the multi-objective optimization performed during the training process, and factors such as distance, angle changes, and step number were incorporated into the reward function, enabling smoother path planning.

In summary, the PM outperforms traditional methods in terms of feasibility, maximum joint deflection angle, time efficiency, and path smoothness. Furthermore, based on the proposed joint coordination motion schemes, the MSR can strictly align with the planned path, enabling precise snake-like motion.

### 5.2. Simulations of Potential Applications

Based on the snake-like motion, the MSR can develop a novel working way. In this section, we demonstrate the potential of applying the MSR to space exploration through two potential applications, involving target capture and robot generation.

**Figure 10:**
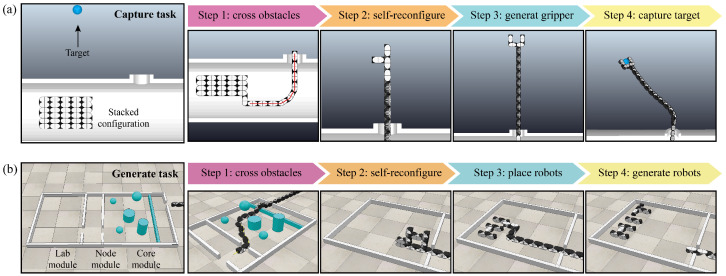
Simulations of potential applications.

The objective of target capture is capturing space debris located outside the space station, thereby preventing potential collision damage to the station. The MSR must traverse a narrow exit hatch and then generate a gripper configuration to achieve the target capture. The process is shown in Figure 10a. Initially, the MSR is folded into a cube structure and placed inside the space station. Upon receiving the task instruction, the robot unfolds its structure to generate propulsion speed and follow the planned path to the exit hatch. Once it reaches the target position, the MSR utilizes the self-reconfiguration method [31] to form a gripper. Finally, it leverages its redundant DOFs to accomplish the target capture. Details can be seen in Movie S1.

While astronauts work inside the space station, various types of robots are often required to assist with repetitive or auxiliary tasks. The MSR is required to navigate through the complex and narrow core module and node module, and it finally reaches the lab module, where it generates various types of robots to meet the task requirements. The process is shown in Figure 10b. Initially, a reasonable path is planned. Then, the MSR utilizes snake-like motion to flexibly traverse complex obstacles and narrow passages. Upon reaching the target position, the MSR utilizes the self-reconfiguration method [31] to sequentially generate three types of robots in the shapes of “H”, “I”, and “T”. Finally, the MSR would utilize its redundant DOFs to places these robots properly for the execution of subsequent tasks. Details can be seen in Movie S2.

In addition, the entire process is reversible, allowing the generated end-effectors and robots to be recycle and reused. This capability significantly enhances resource efficiency, greatly reduces reliance on earth resupply, and offers a novel working way for the future space exploration.

## 6. Conclusions and Prospect

This paper designed a modular snake-like robot based on the module Subot and proposed a motion analysis and planning method to explore a special snake-like motion mode. In the way, modular robots can complete on-site and on-demand reconfiguration, achieving an organic combination of environmental adaptability and versatility. Among them, the procedural framework, analytical basis, joint coordination schemes, constraint resolution methods, and path planning mentioned in the motion analysis and planning method have strong general applicability and can be extended to other types of modular robots. Hence, the study in this paper can lay a theoretical foundation and achieve functional breakthroughs for the application of modular robots in space exploration.

Future work will focus on the hardware experiments to further validate the feasibility and superiority of the MSR. The critical challenge is creating a ground-based microgravity environment. Currently, we are developing a micropore air flotation platform, which utilizes graphite and high-pressure gas to form an air film. Based on the platform, whether the joint force and structural strength can meet the requirements of operation and locomotion will be investigated, which is necessary for the improvement of hardware. Additionally, we will construct a dynamic model for the MSR and explore methods for high-precision interaction, energy optimization, and efficient control, which will advance the applications of the MSR in space exploration.

## Data Availability

The original contributions presented in this study are included in the article/Supplementary Materials. Further inquiries can be directed to the corresponding authors.

## Supplementary Materials

The following supporting information can be downloaded at: https://www.mdpi.com/article/10.3390/biomimetics10050293/s1, Figure S1: Result of MSR moves along planned path in 2-d environment; Figure S2: Result of MSR moves along planned path in 3-d environment; Figure S3: Result of ablation studies; Figure S4: Results of hardware experiments; Table S1: Parameters of Subot; Movie S1: Debris capture; Movie S2: On-site generation of robot swarms; Movie S3: Fold-unfold experiment; Movie S4: Snake-like motion experiment. Reference [38] is cited in the supplementary materials.
