# Supplementary material for: Modular Snake-like Robot Designed for On-Site Reconfiguration in Space Exploration
Source: Biomimetics (Basel). 2025 May 6;10(5):293. doi: 10.3390/biomimetics10050293 (PMC12109486; doi:10.3390/biomimetics10050293)
Supplement: Supplementary file 1 [file biomimetics-10-00293-s001.zip › Supplementary file.pdf]

S1. Simulation settings and results

The simulation environments include two types. In 2-d environment, the map is a square with a side length of 100mm, containing 6 distributed circular obstacles with a radius of 10mm. The positions of obstacles are (42.5, 22), (55, 54), (80, 30), (15, 46), (35, 78), (85, 74), the initial position of agent is (50, 10), and the step length is 5mm. In the specific test, the target position is set to (50, 100), while in the repeated tests, it is randomly assigned to a location at the top of the map. In 3-d environment, the map is a cube with a side length of 100mm. The obstacles are 6 distributed spheres with a radius of 15mm, and positions are (32, 22, 20), (55, 54, 40), (80, 30, 50), (15, 46, 60), (35, 78, 80), (85, 74, 90). The initial position of robot is (50, 50, 10), and the step length is 10mm. In the specific test, the target position is set to (90, 10, 90), while in the repeated tests, it is randomly assigned to a location at the top of the map. Due to the constraints of the rigid linkage, the agent is deemed to have reached the target if it comes within 5mm of the target position.

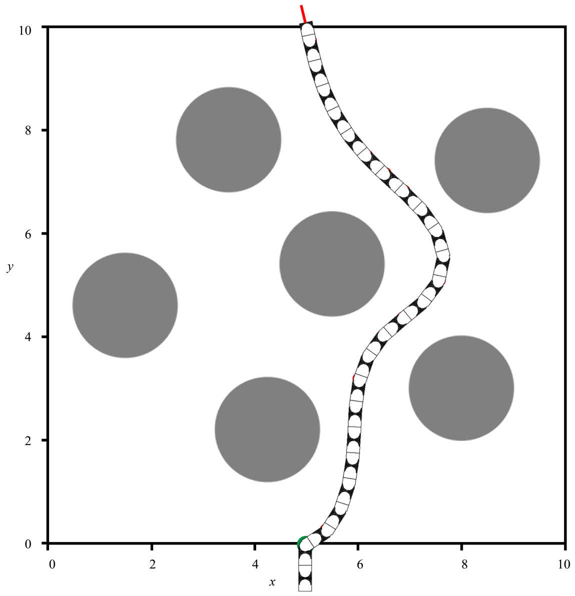

Figure S1. Result of MSR moves along planned path in 2-d environment.

In RRT and MRRT methods, the maximum number of iterations is 10^3 and 10^4 in 2-d and 3-d environment, respectively. The goal bias is 0.05, ensuring a balance between exploration and convergence towards the target. The Euclidean distance is used as the distance metric. The collision checking is performed using distance-based method. The

nearest neighbour search method is implemented using brute-force search. In PM method, the policy and value networks are designed using an architecture of two fully connected layers. Since the experimental environments is relatively simple, the policy network consisted of 16 neurons, while the value network had 32 neurons. This network architecture has the ability to validate the effectiveness of the state, action and reward design, as a complex network architecture does not significantly enhance performance. Both used the ReLU activation function, The model was trained with the Adam optimizer and a learning rate of $3{\times}10^4$. To ensure stable training, the number of steps per update is 512 and batch size is 128. The discount factor is 0.99, and the GAE parameter is 0.95, balancing bias and variance in the advantage estimation. The entropy coefficient i s s et t o 0 .01 a nd 0 .02 to encourage exploration during training. Results of MSR moves along planned path are shown in Figures S1 and S2.

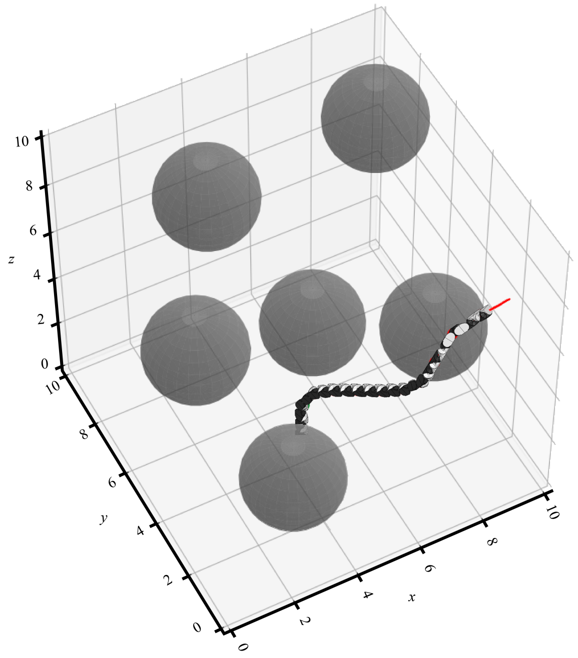

Figure S2. Result of MSR moves along planned path in 3-d environment.

S2. Ablation studies

To further verify the rationality of the path planning algorithm, we conducted ablation studies. In the path planning, the reward consists of four components: distance, collision, deflection angle, and step number. Distance and collision are essential for path planning and cannot be removed. Therefore, ablation studies were conducted on the deflection angle and step number, which utilized the 2-d obstacle environment and resulted in three experiments. In Experiment 1, only distance and collision are utilized as reward for planning. In Experiment 2, the deflection angle is added to the reward for planning. In Experiment 3, the step number is added to the reward for planning.

The results are shown in Figure S3. It can be intuitively observed that while the planned path in Experiment 1 can reach the target position, it lacks smoothness. After adding the deflection angle to reward (Experiment 2), the planned path becomes smooth, but there is a noticeable curvature, which increases the path length. After adding the step number to reward (Experiment 3), the curvature of the path is significantly reduced, showing enhanced performance. Data analysis reveals that Experiment 1 has the highest maximum deflection angle, reaching 59.71°, while Experiment 2 has the smallest at 34.41°, and Experiment 3 is close to Experiment 2 at 36.58°. In terms of steps, Experiment 3 uses 20 steps, which is

fewer than the 22 steps in Experiment 1 and 21 steps in Experiment 2. The total deflection angle in Experiment 1 is 489.43°, which is greater than 248.92° in Experiment 2 and 205.69° in Experiment 3. Although Experiment 3 sacrifices the performance of the maximum deflection angle to reduce the step number, the reduction in steps results in the lowest total deflection angle, making its overall performance superior. This provides a foundation for efficient and energy-saving locomotion of MSR.

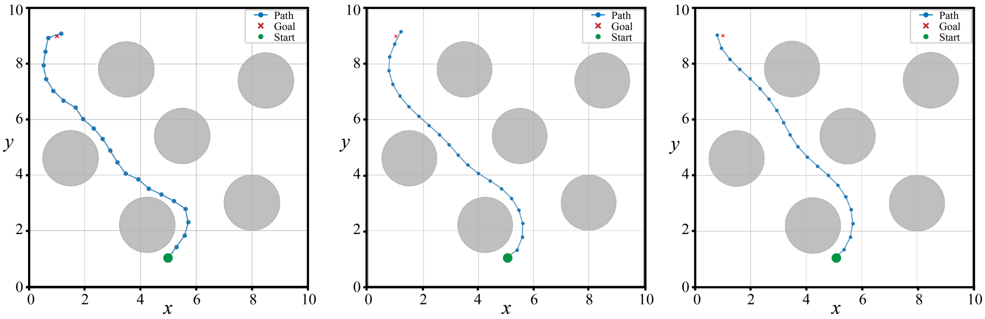

(a) Performance of Experiment 1
t=0.014s, max(α)=59.71°, $\sum$α=489.43°, n=22

(b) Performance of Experiment 2
t=0.016s, max(α)=34.41°, $\sum$α=248.92°, n=21

(c) Performance of Experiment 3
t=0.021s, max(α)=36.58°, $\sum$α=205.69°, n=20

Figure S3. Results of ablation studies.

S3. Hardware experiments

To conduct microgravity experiments in terrestrial environments, we have been developing an air flotation platform for modular robots. The preliminary result is a 1m×1m micropores air flotation platform, which utilizes graphite and high-pressure gas to form an air film. The related work on this platform is currently being submitted to the IEEE/RSJ International Conference on Intelligent Robots and Systems (IROS 2025) [38]. Using this platform and existing module prototypes, we conducted fold-unfold experiment and snake-like motion experiment, verifying the rationality and feasibility of the proposed method. The experiments and results can be seen in Figure S4 and Movies S3 and S4.

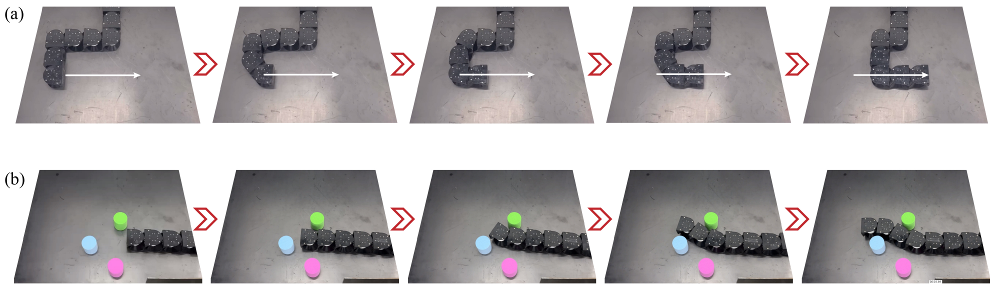

Figure S4. Results of hardware experiments. (a) Fold-unfold experiment. (b) Snake-like motion experiment.

Due to the limitations in the prototype number and the platform size, we conducted experiments with a limited number of modules. In the fold-unfold experiment, MSR can slowly unfold from the folded state and generate a propulsion speed. In the snake-like motion experiment, MSR can avoid obstacles and reach the target. During the experiments, MSR exhibited slight jitter, attributable to two main factors. First, the platform we developed is not yet fully refined, resulting in an unstable air film. Second, each module of MSR rotates at different angles with variable completion times. The current module prototypes lack angle feedback function, so we employed a scheme of delayed command transmission. However, in some situations, certain joints that have completed their rotation should wait

for all modules to finish but instead receive the next command, causing the rotation plan to fail. In the future, we will further optimize these two aspects.

S4. Parameters of Subot

The design parameters related to Subot can be found in Table S1.

Table S1. Parameters of Subot.

Parameters	Values
Dimension(mm)	92×92×92
Weight(g)	520
Encoder chip	MT6816
Driver chip	RZ7899
Joint torque(Nm)	1.75
Range of joint(°)	[-90, 90]
Degrees of freedom	2
Docking surfaces	4
Power capacity(mA)	400
EPM end	Fe
EPM core	AlNiCo and NdFeB
Dimension of EPM(mm)	16×11.34×8.5
Coil diameter(mm)	0.4
Coil turns	80
Average tensile load(N)	96.98
Average shear load(N)	55.15
Maximum bending moment(Nm)	1.68
